# Advancing single-cell RNA-seq data analysis through the fusion of multi-layer perceptron and graph neural network

**DOI:** 10.1093/bib/bbad481

**Published:** 2024-01-03

**Authors:** Xiang Feng, Yu-Han Xiu, Hai-Xia Long, Zi-Tong Wang, Anas Bilal, Li-Ming Yang

**Affiliations:** Department of Information Science Technology, Hainan Normal University, 99 Longkun Road, Haikou, Hainan 571158, China; Department of Information Science Technology, Hainan Normal University, 99 Longkun Road, Haikou, Hainan 571158, China; Department of Information Science Technology, Hainan Normal University, 99 Longkun Road, Haikou, Hainan 571158, China; Department of Pathophysiology, School of Basic Medical Sciences, Harbin Medical University, Harbin 150081, China; Department of Information Science Technology, Hainan Normal University, 99 Longkun Road, Haikou, Hainan 571158, China; Department of Pathophysiology, School of Basic Medical Sciences, Harbin Medical University, Harbin 150081, China

**Keywords:** cell clustering, single-cell RNA-seq, gene imputation, graph neural network, multi-layer perceptron, graph attention network

## Abstract

The advancement of single-cell sequencing technology has smoothed the ability to do biological studies at the cellular level. Nevertheless, single-cell RNA sequencing (scRNA-seq) data presents several obstacles due to the considerable heterogeneity, sparsity and complexity. Although many machine-learning models have been devised to tackle these difficulties, there is still a need to enhance their efficiency and accuracy. Current deep learning methods often fail to fully exploit the intrinsic interconnections within cells, resulting in unsatisfactory results. Given these obstacles, we propose a unique approach for analyzing scRNA-seq data called scMPN. This methodology integrates multi-layer perceptron and graph neural network, including attention network, to execute gene imputation and cell clustering tasks. In order to evaluate the gene imputation performance of scMPN, several metrics like cosine similarity, median L1 distance and root mean square error are used. These metrics are utilized to compare the efficacy of scMPN with other existing approaches. This research utilizes criteria such as adjusted mutual information, normalized mutual information and integrity score to assess the efficacy of cell clustering across different approaches. The superiority of scMPN over current single-cell data processing techniques in cell clustering and gene imputation investigations is shown by the experimental findings obtained from four datasets with gold-standard cell labels. This observation demonstrates the efficacy of our suggested methodology in using deep learning methodologies to enhance the interpretation of scRNA-seq data.

## INTRODUCTION

The introduction of single-cell RNA sequencing (scRNA-seq) technology has made it easier to explore gene expression (GE) within extensive datasets of individual cells, providing robust technological support for such investigations. scRNA-seq is a commonly used technique in cellular research, facilitating the investigation of inter cellular GE and the sequencing of individual genes [1, 2]. Integrating the acquired GE data makes it feasible to derive probable associations among cells, unveil novel cell types and discern cellular heterogeneity [3–5]. As mentioned above, the findings have significant ramifications in detecting and treating intricate medical conditions [6]. Nevertheless, there are some obstacles [7]. One prominent characteristic of scRNA-seq data is the presence of a considerable proportion of zero values, resulting in a substantial sparsity level. The observed zero values in this context may be attributed to either a genuine absence of GE inside the cell or to genes with low expression levels that have not been discovered. These occurrences are sometimes referred to as dropout incidents. The scale of single-cell data continues to grow as high-throughput sequencing technology progresses. The scRNA data exhibit substantial dropout rates due to the low quantities of RNA expression in individual cells. Moreover, the characteristics of single-cell data include high dimensionality, sparsity and noise, which contribute to the complexity and variability of data distributions. Consequently, the analysis of single-cell data becomes more challenging [8].

Various gene imputation methods have been proposed for scRNA-seq data to address the above--mentioned issues. For instance, MAGIC [9] is a Markov Affinity-based denoising algorithm that leverages cell relationships and gene statistics to impute skipping values. DCA [10] is based on neural networks and uses a deep count auto-encoder with a zero-inflated negative binomial loss function to denoise scRNA-seq data and capture non-linear gene–gene relationships. DeepImpute [11] employs multiple sub-neural networks and a divide-and-conquer approach for gene imputation. Meanwhile, scVI [12], scImpute [13] and SAVER [14] utilize deep learning or statistical methods for gene imputation. However, these methods can only treat Euclidean space data, such as expression matrices, and cannot directly be treated with non-Euclidean space data, such as cell graphs. These methods lack effective denoising techniques during the data analysis, leading to less-than-optimal results [15].

Cell clustering is an extremely important part of scRNA-seq data analysis, which can identify unknown cell types and explore the functions of potential tissues. Methods such as SC3 [16], CIDR [17], pcaReduce [18], SIMLR [19] and SHARP [20] reduce the dimensionality of data before clustering by using PCA, UMAP and t-SNE to obtain the low-dimensional representation of data. scDeepCluster [21], scAIDE [22], SCA [23], AAE-SC [24] and scGMAI [25] based on deep learning use auto-encoders to reduce the dimensionality of data, ensuring that the input is equal to the output, and thus obtaining data features. These approaches aim to enhance clustering performance by directly reducing the dimensionality of data. Nevertheless, it’s crucial to acknowledge that these dimensionality reduction methods are sensitive to sequencing platforms, potentially influencing the obtained clustering results. Methods such as SAFE [26] and SAME [27] combine the clustering results of multiple clustering methods to obtain the final clustering results. However, these clustering approaches cannot accurately handle complex and large-scale single-cell data [28].

To better analyse scRNA-seq data, we propose a new single-cell data analysis method called scMPN. This approach utilizes a multi-layer perceptron (MLP)-based encoder and multiple auto-encoder structures to achieve faster and more accurate analysis of single-cell functional data. Additionally, scMPN employs a variation graph auto-encoder to handle non-Euclidean spatial data. By incorporating a graph attention mechanism (GAT) [29], scMPN can effectively analyse and process scRNA-seq data, revealing cell-to-cell relationships, enabling gene imputation and cell clustering, and achieving excellent results that firmly establish a foundation for downstream analysis.

## MATERIALS AND METHODS

### scMPN model

**Figure 1:**
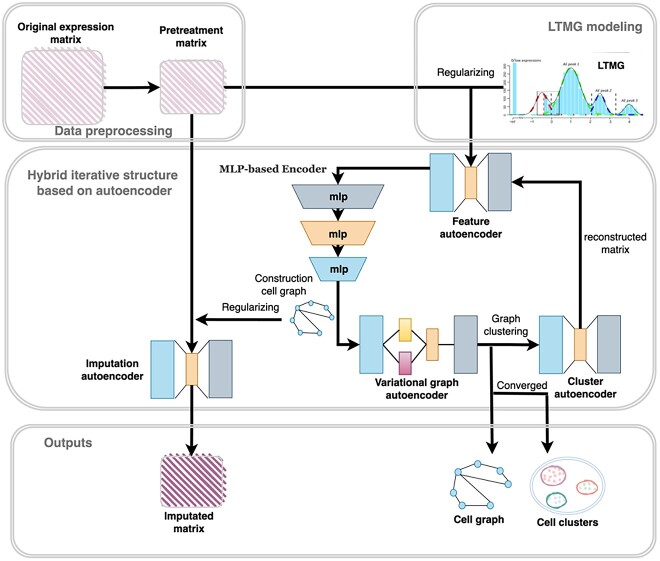
The structure of the scMPN model.

The workflow of the scMPN is depicted in Figure 1, which is constructed based on a graph neural network framework. scMPN integrates a multi-layer MLP-based encoder, multiple auto-encoders and a graph attention network to achieve gene imputation in addition to cell clustering. scMPN primarily consists of a multi-layer MLP neural network used to construct the cell graph and four auto-encoders: a feature auto-encoder for extracting data features, a variation graph auto-encoder (VGAE) for capturing inter-cellular relationships, a cluster auto-encoder for reconstructing GE matrices and an imputation auto-encoder for inferring missing GE values.

scMPN takes a gene expression matrix (GEM) comprising scRNA-seq data as input during the initial iteration. It employs the Left Truncated Mixture Gaussian model (LTMG) as a regularizer to convert the input GE data into discrete regulatory signals. Following training, the feature auto-encoder processes the GEM, extracting embedding from the matrix. The multi-layer MLP-based encoder takes the embedding extracted by the feature auto-encoder as input and further denoises them to generate new embedding. The cell graph is constructed and pruned based on the re-extracted embedding. The pruned cell graph is fed into the variation graph auto-encoder, which optimizes the reconstruction error and KL divergence to prevent over fitting and obtain latent variables as node embedding. The GAT is incorporated to assign different weights to different nodes based on their information, facilitating the exploration of potential cell relationships and inference of cell types. Cell clustering is accomplished by leveraging the be-trained embedding from the cell graph. Every inferred cell type utilizes a specialized cluster auto-encoder to rebuild the GE values. The GEM that has been rebuilt is repeatedly used as input iteratively until convergence is achieved. This iterative process ultimately leads to the generation of cell clustering findings. The imputation auto-encoder utilizes the original GEM as its input. The cell–cell link derived from the pruned cell graph is employed as a means of regularization and imputation of missing GE levels. Through iterative training and embedding of scRNA-seq data into a low-dimensional space, scMPN leverages the topology of the cell graph to recover true GE values and achieve cell clustering.

scMPN demonstrates significant advantages in inferring inter-cellular relationships, including cell type identification, cell trajectory inference and completion of missing gene information based on the acquired cell relationships.

**Figure 2:**
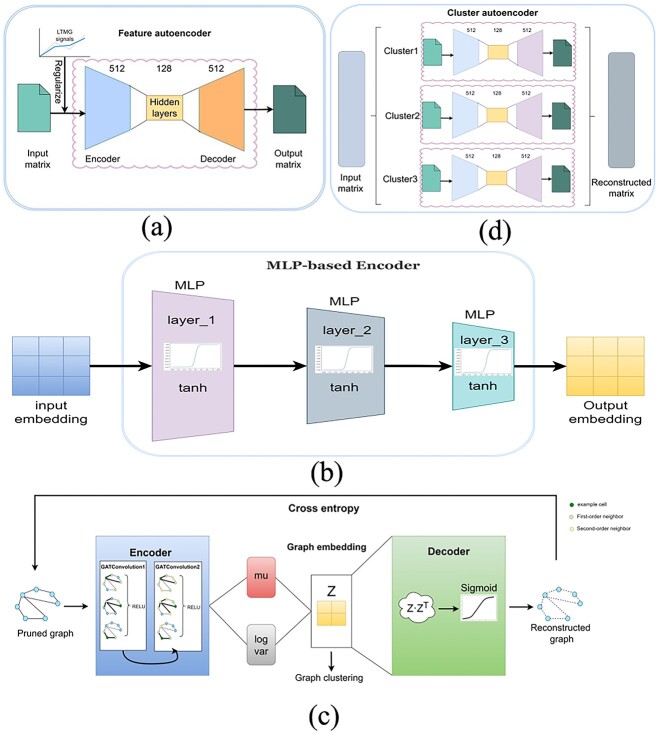
The architecture of the scMPN model is described as follows: (**A**) illustrates the structure of the feature auto-encoder, (**B**) showcases the architecture of the MLP-based encoder, (**C**) depicts the structure of the variation graph auto-encoder and (**D**) outlines the architecture of the cluster auto-encoder.

The architecture of the feature auto-encoder is depicted in Figure 2(A). The original GEM, comprising scRNA-seq data, undergoes sorting based on the standard deviation of genes, and, subsequently, low-quality cells and genes are excluded. The resulting expression matrix, containing the top 2000 genes, serves as the input for the feature auto-encoder. The LTMG model is a regularizer for variable genes. Distinct regulatory states of genes in different cells are treated differently using a loss function. The encoder and decoder of the feature auto-encoder have dimensions of 512 × 128 and 128 × 512, respectively. Training the feature auto-encoder involves minimizing the loss-function to reconstruct the GEM faithfully. Latent embedding is extracted through iterative dimensionality reduction and reconstruction, capturing inter-cellular information.

Figure 2(B) depicts the arrangement of the MLP-based encoder in the system. It consists of a MLP, a neural network architecture that only includes an encoder and lacks a decoder. The MLP-based encoder in this model comprises three layers of MLP structures, and each layer utilizes the Tanh non-linear activation function. This activation function transforms negative inputs into strong negative inputs, while 0 inputs are kept close to 0. The MLP-based encoder takes the embedding output from the feature auto-encoder as input and undergoes multiple layers of MLP structures for multiple dimensionality reduction and denoising. This process effectively extracts cell node information from the input data, resulting in new embedding. The cell graph is formulated using the recently extracted embedding information, with each node representing an individual cell and edges denoting the connections between cells. To enhance the precision of the cell graph, pruning is implemented to eliminate edge noise, and for the K-nearest neighbors graph, an adaptive number of neighbouring nodes is chosen.

Figure 2(c) illustrates the architecture of the variation graph auto-encoder. The encoder of the variation graph auto-encoder consists of twofold layers of graph attention convolution, each equipped with the ReLU activation function. It takes the prune back cell graph as input and learns the mean and variance of the low-dimensional vector representation for each sample through the encoder. It generates random numbers for different features based on the learned mean and variance, preserving the input correlations and constructing a dedicated normal distribution for each sample. The decoder reconstructs the cell graph by utilizing the obtained normal distributions. The goal is to minimize the loss function, ensuring that the reconstructed cell graph closely matches the input. The variation graph auto-encoder incorporates a GAT, enabling the addition of different weights to different nodes and facilitating better graph embedding learning. To optimize model training, the loss function comprises the Reconstruction Loss and the KL-Latent Loss, aligning with a standard normal distribution by minimizing the loss-function. The model is iteratively refined through continuous optimization. Following that, the K-means clustering technique is employed to group the cell nodes within the graph. The Louvain method is then utilized to determine the number of cellular clusters present in the cell graph.

Figure 2(D) outlines the architecture of the cluster auto-encoder, where the input is the GEM reconstructed by the feature auto-encoder. This cluster auto-encoder is responsible for reconstructing the expression matrix for each cell cluster. Leveraging the cell relationships acquired from the variation graph auto-encoder, a distinct auto-encoder is created for each identified cell cluster, allowing tailored treatment for different cell types. Each auto-encoder undergoes individual training, and the concatenated outcomes from all cluster auto-encoders constitute the reconstructed matrix. The iterative training process continues until the cell clustering results stabilize. The rebuilt matrix is inputted into the feature auto-encoder, and the resultant cell clustering is regarded as the ultimate prediction.

After the completion of the iterative procedure, the imputation auto-encoder undertakes the processing of the preprocessed GEM. The model is enhanced by including an L1 regularizer, which considers the generated cell graph and the connections between the cells obtained throughout the iteration process. The regularizer used in this study comprises the edges of the cell graph and the projected cell types. Incorporating the L1 penalty, well known for its intrinsic sparsity, greatly improves the auto-encoder model’s generalization capacity. In conclusion, the imputation auto-encoder generates the reconstructed GE levels as the outcome of the imputation process.

### Data preprocessing and normalization

The scMPN model processes a GEM derived from scRNA-seq data as input, facilitating robust single-cell data analysis through cell clustering and gene imputation. However, challenges arise from the high dropout rate inherent in scRNA-seq data. Only a small fraction of genes, exceeding 1%, are expressed in cells, with more than 1% retaining non-zero GE. Therefore, handling and filtering scRNA-seq data are particularly important. Genes are classified based on their standard deviation, and the top 2000 genes are selected for analysis. All data are normalized through a logarithmic transformation. The preprocessed GEM is then input into the scMPN. The LTMG model is used to regularize the top 2000 variable genes, inferring the distribution and state of the single-GE profile. Low-expressed and missing genes are modeled as left-truncated data with finite experimental resolution. The LTMG model processes the GEM, exploring different cells’ GE regulatory signals in scRNA-seq data. Based on the dynamics between transcriptional regulation input, mRNA metabolism and abundance, the model exhibits a significantly better fit on a large-scale scRNA-seq data set. LTMG, as a high-order constrained regularization feature auto-encoder, imparts a higher signal-to-noise ratio to the explored regulatory signals. When the number of cells is *N*, gene countenance values are represented as $\mathrm{X}=\left\{{\mathrm{x}}_1,{\mathrm{x}}_2,\cdots, {\mathrm{x}}_{\mathrm{n}}\right\}$, where each ${\mathrm{x}}_{\mathrm{j}}\in$X. The expression of gene X is modeled by a combination of K Gaussian distributions, each representing a potential gene regulatory signal (TRS). The density function of gene X may be described as follows:


(1) \begin{align*} \mathrm{\rho} \left(\mathrm{X};\mathrm{\theta} \right)&=\prod_{\mathrm{j}=1}^{\mathrm{N}}\mathrm{\rho} \left({\mathrm{x}}_{\mathrm{j}};\mathrm{\theta} \right)=\prod_{\mathrm{j}=1}^{\mathrm{N}}\sum_{\mathrm{i}=1}^{\mathrm{k}}{\mathrm{\alpha}}_{\mathrm{i}}\mathrm{\rho} \left({\mathrm{x}}_{\mathrm{j}};{\mathrm{\theta}}_{\mathrm{i}}\right)\nonumber\\&=\prod_{\mathrm{j}=1}^{\mathrm{N}}\sum_{\mathrm{i}=1}^{\mathrm{k}}{\displaystyle \begin{array}{c}{\mathrm{\alpha}}_{\mathrm{i}}\frac{1}{\sqrt{2\mathrm{\pi} {\mathrm{\sigma}}_{\mathrm{i}}}}{\mathrm{e}}^{\frac{-{\left({\mathrm{x}}_{\mathrm{j}}-{\mathrm{\mu}}_{\mathrm{i}}\right)}^2}{2{\mathrm{\sigma}}_{\mathrm{i}}^2}}=\mathrm{L}\left(\mathrm{\theta}; \mathrm{X}\right)\ \\{}\ \end{array}} \end{align*}

In the LTMG model, $\alpha_{\rm i}$ represents the mixture probability, $\mu_{\rm i}$ indicates the mean value of the *i*th Gaussian distribution and $\sigma_{\rm i}$ represents the standard deviation. The parameter θ is derived using the EM algorithm, which characterizes the error for simulated zero and low-expression values. Operating under the left-truncation assumption, the GE profile is decomposed into two components: M, representing the expression of truly measured values, and N-M, indicating the GE truncated on the left side under N conditions. Maximizing the parameter is achieved using the likelihood function, and an expectation–maximization method may accomplish its estimation. The Bayesian Information Criterion determines the optimal number of Gaussian components. Following that, the initial GE levels are allocated to the distribution that is deemed most probable for each cell. The formulation of the likelihood of belonging to distribution *i* is described in depth as


(2) \begin{equation*} \mathrm{p}\left({\mathrm{x}}_{\mathrm{j}}\in \mathrm{TRS}\ \mathrm{i}|\mathrm{K},\mathrm{\theta} \ast \right)\mathrm{\varepsilon} \frac{\alpha_{\mathrm{i}}}{\sqrt{2\mathrm{\pi} {\mathrm{\sigma}}_{\mathrm{j}}^2}}{\mathrm{e}}^{\frac{-{\left({\mathrm{x}}_{\mathrm{j}}-{\mathrm{\mu}}_{\mathrm{i}}\right)}^2}{2{\mathrm{\sigma}}_{\mathrm{i}}^2}} \end{equation*}

where ${\mathrm{x}}_{\mathrm{j}}$ is labeled by$\mathrm{TRS}\ \mathrm{i}$ if $\mathrm{p}({\mathrm{x}}_{\mathrm{j}}\in \mathrm{TRS}\ \mathrm{i}|\mathrm{K},\mathrm{\theta} \ast )=\underset{\mathrm{i}=1.\dots \mathrm{k}}{\max}(\mathrm{p}({\mathrm{x}}_{\mathrm{j}}\in \mathrm{TRS}\\mathrm{i}|\mathrm{K},\mathrm{\theta} \ast ))$. Thus, each gene’s discrete values (1,2, …, K) are generated.

### Feature auto-encoder

The auto-encoder feature comprises two main components: an encoder and a decoder. The input for the procedure is the processed GEM, which undergoes regularization processing through the LTMG model to obtain a representative embedding of the single-cell RNA expression. The encoder produces a low-dimensional representation using the input GEM, while the decoder reconstructs the GEM based on the acquired embedding. The primary objective in training the feature auto-encoder is to minimize the loss function, ensuring that the reconstructed GEM closely mirrors the input GEM. The (MSE) is employed to assess the contrast between the reconstructed GEM ($ \hat{\text{X}} $) and the input GEM (X) and is defined as follows:


(3) \begin{equation*} \sum{\left(\mathrm{X}-\hat{\mathrm{X}}\right)}^2 \end{equation*}

### MLP-based encoder

The MLP-based encoder is a neural network model consisting of multiple MLP layers. It takes the extracted embedding as input and employs the Tanh activation function at each layer to address non-linearity and enhance the model’s expressive power. After successive MLP neural network layers perform dimensionality reduction and denoising, new embedding is extracted to construct the cell graph. If the input is denoted as X, where the Tanh activation function is given by


(4) \begin{equation*} \mathrm{Tanh}\left(\mathrm{X}\right)=\frac{{\mathrm{e}}^{\mathrm{X}}-{\mathrm{e}}^{-\mathrm{X}}}{{\mathrm{e}}^{\mathrm{X}}+{\mathrm{e}}^{-\mathrm{X}}} \end{equation*}

In this equation, the Tanh function maps the input *X* to a range between −1 and 1, allowing the MLP-based encoder to model complex non-linear relationships in the data. If we denote the input of the MLP-based encoder at layer *i* as *i* and represent the output of layer *i* as $\mathrm{h}\left({\mathrm{x}}_{\mathrm{i}}\right)$, the expression for $\mathrm{h}\left({\mathrm{x}}_{\mathrm{i}}\right)$ can be defined as follows in the context of the MLP-based encoder:


(5) \begin{equation*} \mathrm{h}\left({\mathrm{X}}_{\mathrm{i}}\right)=\mathrm{Tanh}\left({\mathrm{W}}_{\mathrm{i}}\ast{\mathrm{X}}_{\mathrm{i}}+{\mathrm{b}}_{\mathrm{i}}\right) \end{equation*}

In this equation, ${\mathrm{W}}_{\mathrm{i}}$ represents the weight matrix associated with layer *i*, ${\mathrm{b}}_{\mathrm{i}}$ is the bias vector, and the Tanh function is applied element-wise to the linear combination of the input *X* with the weights and biases of layer *i*. The resulting output $\mathrm{h}\left(\mathrm{x}\right)$ is a transformed representation that captures the non-linear relationships and higher-level features learned by the MLP-based encoder at layer *i*.

### Variation graph auto-encoder

The variation graph auto-encoder is a deep-learning model that uses graph attention, specifically designed to handle sparse matrix data by incorporating layers of graph attention. Taking the cell graph as input, it employs the encoder to compute the mean vector and covariance matrix, from which samples are drawn to obtain the embedding. The decoder then reconstructs the cell graph based on the learned embedding. Two graph attention convolution layers are added to the encoder to enhance the model’s ability to capture cell relationships effectively. The computation process of the graph attention convolution layer is as follows:


(6) \begin{align*} &\mathrm{GraphAttention}\left({\mathrm{H}}^{\left(\mathrm{l}\right)},\mathrm{A}\right)\nonumber\\&=\mathrm{softmax}\left(\mathrm{LeakyReLU}\left(\overset{\sim }{\mathrm{A}}{\mathrm{W}}^{\left(\mathrm{l}\right)}\right){\overset{\sim }{\mathrm{H}}}^{\left(\mathrm{l}\right)}{\mathrm{W}}^{\left(\mathrm{l}\right)}\right) \end{align*}

In this equation, ${\mathrm{H}}^{(1)}$represents the input feature matrix at layer l. A is the adjacency matrix of the graph, ${\mathrm{W}}^{(1)}$ indicates the weight matrix of layer l, $\overset{\sim }{\mathrm{A}}$ is the attention coefficients matrix obtained by applying the softmax function to the output of the Leaky ReLU activation function and ${\overset{\sim }{\mathrm{H}}}^{(1)}$ represents the normalized attention coefficients matrix multiplied by the input feature matrix. This process calculates the attention weights for each node in the graph based on its neighbouring nodes and updates the features accordingly. The output dimensions for the first as well as second levels are 32 along with 16, respectively, with a learning rate set to 0.001. The variation graph auto-encoder utilizes a graph attention network to compute the mean and variance, representing the mean and contrast of node vector representations. An attention mechanism is introduced to enhance the capturing of information from individual units, assigning weights to neighbouring nodes. This addition enables the variation graph auto-encoder to learn the embedding of the cell graph more effectively. The formula for computing attention coefficients is as follows:


(7) \begin{equation*} {\mathrm{e}}_{\mathrm{i}\mathrm{j}}=\mathrm{a}\left(\overrightarrow{{\mathrm{h}}_{\mathrm{i}}},\overrightarrow{{\mathrm{h}}_{\mathrm{j}}}\right)=\mathrm{W}\overrightarrow{{\mathrm{h}}_{\mathrm{i}}}\bullet \mathrm{W}\overrightarrow{{\mathrm{h}}_{\mathrm{j}}} \end{equation*}

The formula denotes the attributes of nodes i and j as $\overrightarrow{h_{\mathrm{i}}}$ and $\overrightarrow{h_{\mathrm{j}}}$, respectively. The attention coefficient between node i and node j, denoted as W, represents the shared weight matrix. The incorporation of multiple independent attention coefficients allows for the extension of a multi-head attention mechanism. Incorporating the average calculation derived from adaptive attention coefficients enhances the stability of the learning process for both unit graph embedding and topological information. The following formula provides the mathematical expression representing the multi-head attention mechanism:


(8) \begin{equation*} {\overrightarrow{\mathrm{h}}}^{\prime }=\mathrm{\sigma} \left(\frac{1}{\mathrm{K}}\sum_{\mathrm{k}=1}^{\mathrm{K}}\sum_{\mathrm{K}\mathrm{\varepsilon} {\mathrm{N}}_{\mathrm{i}}}{\mathrm{a}}_{\mathrm{i}\mathrm{j}}^{\mathrm{k}}{\mathrm{W}}^{\mathrm{k}}\overrightarrow{{\mathrm{h}}_{\mathrm{i}}}\right) \end{equation*}

In a variation graph auto-encoder, the encoder computes the final output by performing a linear transformation of the mean vector obtained through calculations and the element-wise multiplication of the standard deviation vector and a randomly sampled vector. The encoder’s output is given by


(9) \begin{equation*} \mathrm{Z}=\mathrm{\mu} +\mathrm{\sigma} \bigodot \mathrm{\varepsilon} \end{equation*}

where *Z* represents the encoder’s output, μ indicates the mean vector, σ represents the standard deviation vector and ϵ represents the randomly sampled vector from a standard normal distribution. In the decoder, we incorporate the sigmoid activation function. The decoder of variation graph auto-encoder is stated as follows:


(10) \begin{equation*} \hat{\mathrm{A}}=\mathrm{sigmoid}\left(\mathrm{Z}{\mathrm{Z}}^{\mathrm{T}}\right) \end{equation*}

The reconstructed adjacency matrix of matrix A is symbolized as $\hat{\mathrm{A}\ }$, while Z signifies the embedding obtained from the encoder. The main aim of the variation graph auto-encoder is to minimize the disparity between the input matrix A and the reconstructed matrix $\hat{\mathrm{A}\ }$. This goal is achieved through the minimization of the cross-entropy L:


(11) \begin{equation*} \mathrm{L}\left(\mathrm{A},\hat{\mathrm{A}}\right)=-\frac{1}{\mathrm{N}\times \mathrm{N}}\sum_{\mathrm{i}=1}^{\mathrm{N}}\sum_{\mathrm{j}=1}^{\mathrm{N}}\left({\mathrm{a}}_{\mathrm{i}\mathrm{j}}\ast \log \left({\hat{\mathrm{a}}}_{\mathrm{i}\mathrm{j}}\right)+\left(1-{\mathrm{a}}_{\mathrm{i}\mathrm{j}}\right)\ast \log \left(1-{\hat{\mathrm{a}}}_{\mathrm{i}\mathrm{j}}\right)\right) \end{equation*}

In this context, ${\mathrm{a}}_{\mathrm{ij}}$and ${\hat{\mathrm{a}}}_{\mathrm{ij}}$ refer to the elements situated at row i and column j in matrix A and the reconstruction matrix $\hat{\mathrm{A}}$, respectively. *N* represents the number of nodes in the cell graph, and given that the cell graph comprises N cells, the total number of elements in the matrix is determined by N × N.

### Iterative process

Through iterations, the obtained embedding can be applied to reconstruct a cell graph that better captures cell relationships, making the graph more biologically meaningful. The iteration process of the cell graph is as follows:


(12) \begin{equation*} \overset{\sim }{\mathrm{A}}=\mathrm{\mu} {\mathrm{L}}_0+\left(1-\mathrm{\mu} \right)\frac{{\mathrm{A}}_{\mathrm{ij}}}{\sum_{\mathrm{j}}{\mathrm{A}}_{\mathrm{ij}}} \end{equation*}

The equation involves the use of ${\mathrm{L}}_0$, which represents the adjacency matrix of the trimmed cell graph. Additionally, μ is a parameter used to modify the pace of the iteration process. The symbol $\overset{\sim }{\mathrm{A}}$ represents the symmetric normalized adjacency matrix, whereas ${\mathrm{A}}_{\mathrm{ij}}$ denotes the specific element located at row i along with column j inside the adjacency matrix A. During the iteration process, two indicators can be used to determine whether the iteration process has converged: (i) checking if the reconstructed adjacency matrix is the same as the previous iteration’s adjacency matrix and (ii) evaluating the similarity of cell types compared to the cell types inferred in the previous iteration using a similarity measure such as Adjusted Rand Index (ARI). The cell clustering outcomes obtained in the previous iteration are subsequently considered the final cell clustering results.

### Imputation auto-encoder

Upon the conclusion of the iteration process, the imputation auto-encoder takes the original GEM from scRNA-seq data as input. It incorporates the constructed cell graph to regularize the GEM. The regularization of the cell graph is explained as follows:


(13) \begin{equation*} {\mathrm{\gamma}}_1\sum \left(\mathrm{A}\bullet{\left(\mathrm{X}-\hat{\mathrm{X}}\right)}^2\right) \end{equation*}

A represents the adjacency matrix obtained in the preceding iteration, and the dot symbol denotes the matrix product. The penalty formula applied to the leading edge of the cell graph during the training process is articulated as follows:


$$ {\mathrm{\gamma}}_2\sum \left(\mathrm{B}\bullet{\left(\mathrm{X}-\hat{\mathrm{X}}\right)}^2\right) $$

(14) \begin{equation*} {\mathrm{B}}_{\mathrm{ij}}=\left\{\begin{array}{c}1\ \mathrm{i}\ \mathrm{and}\ \mathrm{j}\ \mathrm{belong}\ \mathrm{to}\ \mathrm{the}\ \mathrm{same}\ \mathrm{cell}\ \mathrm{type}\\{}0\ \mathrm{else}\ \end{array}\right. \end{equation*}

The connection matrix, denoted as B, represents the cellular associations. When the cell types recognized by two cells are the same, the corresponding entry in the matrix is assigned a value of 1. Conversely, if the cell types differ, the entry is assigned a value of 0. ${\mathrm{\gamma}}_1$,${\mathrm{\gamma}}_2$ is the intensity of regularization. The definition of L1 regularizer as the regularizer of imputation auto-encoder is as follows:


(15) \begin{equation*} \mathrm{\beta} \sum \left|\mathrm{w}\right| \end{equation*}

In this context, the variable ‘w’ denotes the weight, whereas the symbol $\sum \left|\mathrm{w}\right|$ signifies the summation of the fixed values of every single element. Minimizing $\mathrm{w}$ enhances the model’s generalization ability while also increasing sparsity. $\mathrm{\beta} \mathrm{\varepsilon} \left[0,1\right]$ represents the strength of L1 regularization. The loss function of the imputation auto-encoder is defined as follows:


(16) \begin{align*} \mathrm{Loss}=\left(1-\mathrm{\alpha} \right)\sum{\left(\mathrm{X}-\hat{\mathrm{X}}\right)}^2+\mathrm{\alpha} \sum \left({\left(\mathrm{X}-\hat{\mathrm{X}}\right)}^2\ast \mathrm{TRS}\right)\nonumber\\+\mathrm{\beta} \sum \left|\mathrm{w}\right|+{\mathrm{\gamma}}_1\sum \left(\mathrm{A}\bullet{\left(\mathrm{X}-\hat{\mathrm{X}}\right)}^2\right)+{\mathrm{\gamma}}_2\sum \left(\mathrm{B}\bullet{\left(\mathrm{X}-\hat{\mathrm{X}}\right)}^2\right) \end{align*}

### Evaluation metrics

To assess the gene imputation capability of scMPN, we introduce dropout into the preprocessed scRNA-seq data, randomly converting 10% of the non-zero values to zero to simulate dropout events. This study employs three imputation metrics: median L1 distance, cosine similarity and root mean squared error (RMSE), calculated using formulas (17–19). Here, X represents the row vector containing the original expression, and Y is the row vector of the imputed expression. Lower values for these metrics indicate better imputation performance.

For evaluating the clustering performance of scMPN, seven metrics are computed: ARI, Adjusted Mutual Information (AMI), Normalized Mutual Information (NMI), Homogeneity Score (HS), Completeness Score (CS), V-Measure Score (VMS) and Fowlkes–Mallows Score (FMS). Higher values for these metrics indicate superior clustering performance.

L1 distance measures the absolute deviation involving the original and estimated data. It is a metric to quantify the differences between imputed GE and ground truth values. A smaller L1 distance value indicates a smaller discrepancy between the computed and true imputed values, suggesting a more accurate imputation:


(17) \begin{equation*} \mathrm{L}1\ \mathrm{distance}=\left|\mathrm{X}-\mathrm{Y}\right| \end{equation*}

The dot product between the original and estimated data determines cosine similarity. It serves as a measure of similarity between imputed values and the ground truth values. A higher cosine similarity value indicates a stronger resemblance between the imputed and true profiles, signifying a closer match between the computed and actual imputed values.


(18) \begin{equation*} \mathrm{cosine}\ \mathrm{similarity}\left(\mathrm{X},\mathrm{Y}\right)=\frac{\mathrm{X}{\mathrm{Y}}^{\mathrm{T}}}{\left\Vert \mathrm{X}\right\Vert \left\Vert \mathrm{Y}\right\Vert } \end{equation*}

RMSE is the square root of the average of the squared differences between the original and estimated data points. It serves as a metric to assess the accuracy of imputed GE values compared to the ground truth values. A smaller RMSE value indicates that the imputation method produces predictions closer to the true values, reflecting higher accuracy in the imputation process:


(19) \begin{equation*} \mathrm{RMSE}\left(\mathrm{X},\mathrm{Y}\right)=\sqrt{\frac{\sum_{\mathrm{i}=1}^{\mathrm{N}}{\left({\mathrm{X}}_{\mathrm{i}}-{\mathrm{Y}}_{\mathrm{i}}\right)}^2}{\mathrm{N}}} \end{equation*}

The similarity between the current clustering results and the actual labels is assessed by computing the ARI:


(20) \begin{equation*} \mathrm{ARI}=\frac{\mathrm{RI}-\mathrm{E}\left[\mathrm{RI}\right]}{\max \left(\mathrm{RI}\right)-\mathrm{E}\left[\mathrm{RI}\right]} \end{equation*}

The RI is defined as


(21) \begin{equation*} \mathrm{RI}=\frac{\mathrm{a}+\mathrm{b}}{{\mathrm{C}}_{\mathrm{n}}^2} \end{equation*}

The formula utilizes the variables a and b to, respectively, denote the count of accurately classified cells within the same set and across various sets. The denominator of the calculation indicates the total number of potential pairings.

AMI, similar to ARI, incorporates information entropy. A higher AMI implies a higher similarity:


(22) \begin{equation*} \mathrm{AMI}\left(\mathrm{x},\mathrm{y}\right)=\frac{\mathrm{MI}\left(\mathrm{x},\mathrm{y}\right)-\mathrm{E}\left(\mathrm{MI}\left(\mathrm{x},\mathrm{y}\right)\right)}{\mathrm{Avg}\left(\mathrm{H}\left(\mathrm{x},\mathrm{y}\right)\right)-\mathrm{E}\left(\mathrm{MI}\left(\mathrm{x},\mathrm{y}\right)\right)} \end{equation*}

The variables x and y are used to denote the inferred as well as standard clustering outcomes, respectively. The variable H denotes the quantity of indeterminate clusters inside a partition set, and its definition is as follows:


(23) \begin{equation*} \mathrm{H}\left(\mathrm{x}\right)=\sum_{\mathrm{i}=1}^{\left|\mathrm{x}\right|}\mathrm{P}\left(\mathrm{i}\right)\log \left(\mathrm{P}\left(\mathrm{i}\right)\right) \end{equation*}

where $\mathrm{P}\left(\mathrm{i}\right)=\left|{\mathrm{x}}_{\mathrm{i}}\right|/\mathrm{N}$, MI is defined as


(24) \begin{equation*} \mathrm{MI}\left(\mathrm{x},\mathrm{y}\right)=\sum_{\mathrm{i}=1}^{\left|\mathrm{x}\right|}\sum_{\mathrm{j}=1}^{\left|\mathrm{y}\right|}\mathrm{P}\left(\mathrm{i},\mathrm{j}\right)\log \left(\frac{\mathrm{P}\left(\mathrm{i},\mathrm{j}\right)}{\mathrm{P}\left(\mathrm{i}\right)\mathrm{P}\left(\mathrm{j}\right)}\right) \end{equation*}

NMI is a modified version of mutual-information (MI). It is expressed as


(25) \begin{equation*} \mathrm{NMI}\left(\mathrm{x},\mathrm{y}\right)=\frac{\mathrm{MI}\ \left(\mathrm{x},\mathrm{y}\right)}{\mathrm{Mean}\left(\mathrm{H}\left(\mathrm{x}\right),\mathrm{H}\left(\mathrm{y}\right)\right)} \end{equation*}

H (x) and H (y) are the entropy of x and y, respectively.

HSand CS are two indicators used to evaluate clustering results, which can be used to measure whether the clustering algorithm divides the data into ‘homogeneous’ (i.e., the cluster only contains sample points of the same category) and ‘complete’ (i.e., whether sample points of the same category are divided into the same cluster):


(26) \begin{equation*} \mathrm{HS}=1-\frac{\mathrm{H}\left(\mathrm{C}/\mathrm{K}\right)}{\mathrm{H}\left(\mathrm{C}\right)} \end{equation*}

(27) \begin{equation*} \mathrm{CS}=1-\frac{\mathrm{H}\left(\mathrm{K}/\mathrm{C}\right)}{\mathrm{H}\left(\mathrm{K}\right)} \end{equation*}

The VMS is a harmonic mean computed by Eq. (28):


(28) \begin{equation*} \mathrm{VMS}=\frac{\left(1+\mathrm{\beta} \right)\times \mathrm{HS}\times \mathrm{CS}}{\mathrm{\beta} \times \mathrm{HS}+\mathrm{CS}} \end{equation*}

where the default value of $\mathrm{\beta}$ is 1.

FMS is the geometric mean of precision and recall, with a value range of [0, 1]:


(29) \begin{equation*} \mathrm{FMS}=\frac{\mathrm{TP}\ }{\sqrt{\left(\mathrm{TP}+\mathrm{FP}\right)+\left(\mathrm{TP}+\mathrm{FN}\right)}} \end{equation*}

## RESULTS AND DISCUSSION

To evaluate the gene imputation and cell clustering performance of scMPN, a comparative analysis was conducted against five other methods: scVI, scIGANs, scImpute, DCA and DeepImpute. The assessment involved four high-quality annotated scRNA-seq datasets. These openly accessible datasets were sourced from the Gene Expression Omnibus (GEO) databases, with the accession numbers GSE75688 (Chung data), GSE65525 (Klein data), GSE60361 (Zeisel data) and E-MTAB-2600 (Kolodziejczy data) obtained from the European Bioinformatics Institute (EMBL-EBI).

### The results of gene imputation

In the present experimental configuration, we replicated the dropout phenomena by randomly transforming non-zero values into zero, implementing dropout rates of 10 and 30%. Three assessment criteria were used for imputation: cosine similarity, median L1 distance, and (RMSE. The performance of the scMPN approach was assessed by comparing it with five other methods, namely scVI, scIGANs, scImpute, DCA and DeepImpute. This comparison was based on a set of assessment metrics.

**Figure 3:**
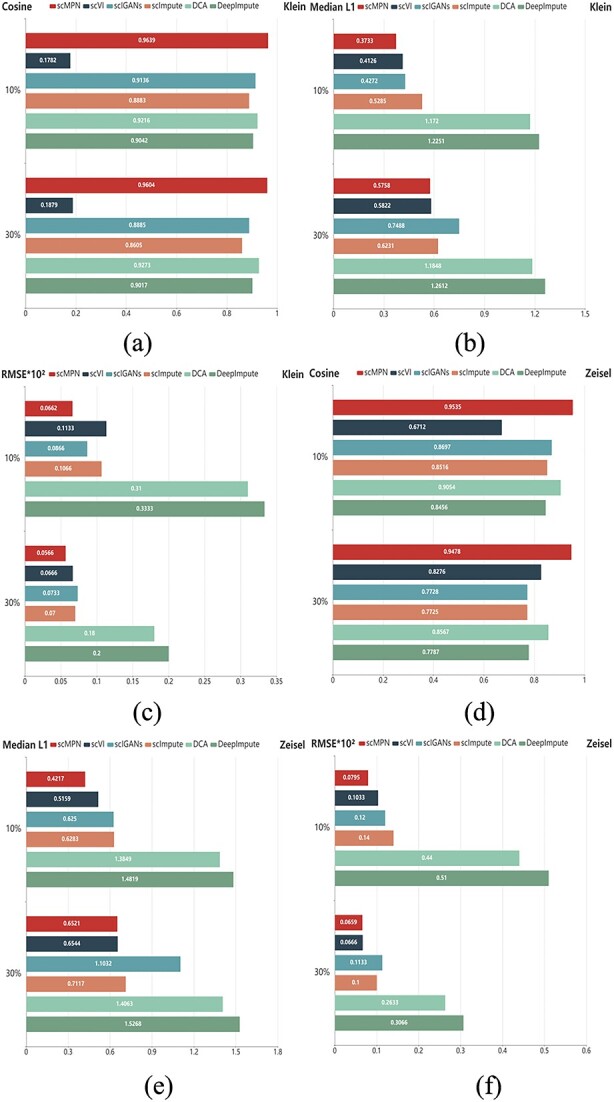
When the dropout rate is 10 and 30%, the gene interpolation performance of the cosine similarity, median L1 distance and RMSE score between the scMPN model and the five existing interpolation methods are compared. (**A**), (**B**) and (**C**) are the results of the Klein datasets. (**D**), (**E**) and (**F**) are the results of the Zeisel datasets.

Figure 3 presents the comparison of cosine similarity (Cosine), median L1 distance (Median L1) and root mean squared error (RMSE) obtained by scMPN and five other methods in gene imputation experiments, with dropout rates set at 10 and 30%, using the Klein (Figures 3A, B, C) and Zeisel (Figure 3D, E, F) datasets. Figure 3 shows that scMPN achieves the best results in all three imputation evaluation metrics at both dropout rates. Regarding cosine similarity in the Klein datasets (Figure 3A), scMPN achieves 0.9639 and 0.9604 at 10 and 30% dropout rates, respectively, outperforming the other five methods. Notably, scVI performs relatively poorly. In the L1 distance median results shown in Figure 3(B), scMPN achieves 0.3733 and 0.5758, outperforming the second-ranked scVI (0.4126 and 0.5822). In the RMSE measured in Figure 3(C), scMPN consistently achieves the best result (0.0662, 0.0566). The results of the Zeisel datasets (Figure 3D, E, F) also indicate that scMPN outperforms the other five methods in terms of imputation performance.

### The results of cell clustering

**Figure 4:**
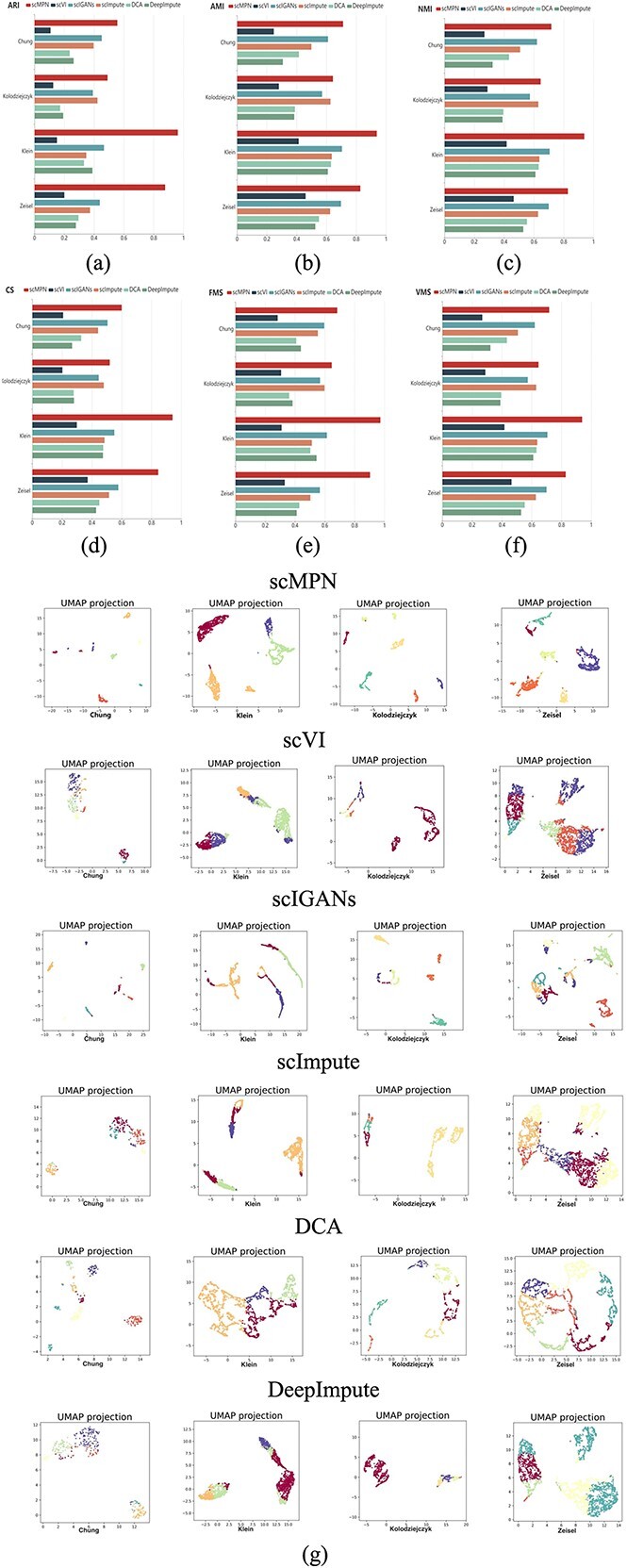
Compare the cell clustering performance of ARI (**A**), AMI (**B**), NMI (**C**), CS (**D**), FMS (**E**) and VMS (**F**), measured by scMPN and five other models using four data sets. (**G**) The UMAP method visualizes the cell clustering results of scMPN and five other models on four datasets.

Cell clustering is a crucial phase in the analysis of scRNA-seq data. Therefore, we conducted experiments to assess the cell clustering performance of scMPN. In Figure 4(A, B, C, D, E, F), we present comparative results of cell clustering achieved by scMPN and five other methods. The evaluation metrics include ARI, AMI, NMI, CS, FMS and VMS. These assessments were conducted on four datasets (Chung, Kolodziejczy, Klein and Zeisel). Figure 4 shows that scMPN achieves the best results in all six clustering evaluation metrics among the six methods. Particularly, the computed clustering metrics in the Klein datasets are significantly superior to those of the second-ranked method, indicating excellent performance. On the other hand, scVI yields the poorest clustering results. These findings demonstrate that scMPN exhibits strong clustering capabilities.

Figure 4(G) displays the visualization results obtained by applying the UMAP method to the four datasets using scMPN and five other cell clustering methods. The scMPN model exhibits tighter clustering of cells contained by the same cluster and higher partition between different clusters than the other five models. In other words, the scMPN model achieves better cell clustering functionality more intuitively.

### Analysis of variation graph auto-encoder mechanism of the scMPN model

The VGAE has proven to be crucial in the cell clustering process. Consequently, we conducted experiments to assess the influence of utilizing or not utilizing VGAE on the clustering results. We tested seven clustering evaluation metrics, ARI, AMI, NMI, CS, FMS, VMS and HS, on the Chung, Kolodziejczy, Klein and Zeisel datasets. Since the VGAE did not directly participate in the imputation process, we did not include imputation as a comparative experiment.

**Figure 5:**
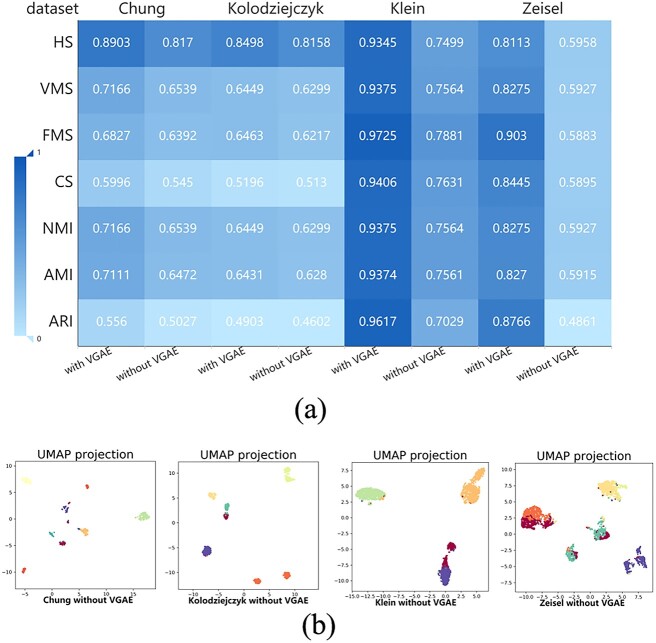
(**A**) Using four datasets, compares seven clustering evaluation metrics, namely ARI, AMI, NMI, CS, FMS, VMS) and HS, for scMPN with and without VGAE (**B**) utilizes the UMAP method for visualizing the cell clustering results obtained by scMPN without VGAE on four datasets.

We compiled the clustering evaluation metrics from the four datasets into a heat map. Figure 5a shows that when scMPN utilizes VGAE, the seven clustering metrics measured on the four datasets are consistently improved compared to when VGAE is not used. Particularly, Chung, Kolodziejczy, Klein, Zeisel. Chung has 317 cells, Kolodziejczy has 704 cells, Klein has 2717 cells and Zeisel has 3005. In these four data sets, Klein and Zeisel belong to larger data sets. Experiments have also proven that using VGAE can obtain better clustering performance in larger datasets Klein and Zeisel when VGAE is employed. Additionally, we visualized the clustering results obtained without using VGAE by the UMAP tool. As depicted in Figure 5(B), compared to the clustering visualization without VGAE in Figure 4(G), the visualization with VGAE exhibits tighter connections among cells contained by the same cluster and greater separation between different clusters.

### Analysis of GAT of the scMPN model

**Figure 6:**
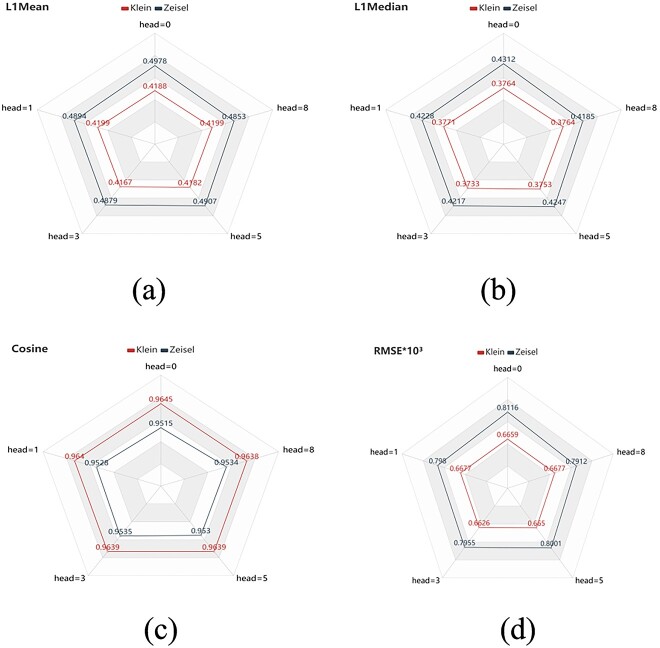
Compares the imputation performance of scMPN devoid of graph attention (head = 0) and with varying levels of graph attention (head = 1, head = 3, head = 5, head = 8) based on evaluation metrics L1Mean (**A**), L1Median (**B**), Cosine similarity (**C**) and RMSE (**D**).

The multi-head attention mechanism plays a significant role in the scMPN model, allowing for assigning different weights to individual cells, thereby facilitating clustering and imputation. We conducted comparative experiments with and without graph attention to investigate the impact of graph attention on imputation performance. Figure 6 presents the results of scMPN with different numbers of attention heads: 0 (no graph attention), 1, 3, 5 and 8, in terms of four imputation evaluation metrics: L1Mean (Figure 6A), L1Median (Figure 6B), Cosine (Figure 6C) and RMSE (Figure 6d). Overall, when head = 3, the best imputation results were observed on the Klein datasets. For the Zeisel data set, the optimal values for L1Mean, L1Median, Cosine and RMSE were achieved with head = 8, head = 8,head = 3 and head = 8, respectively. A single attention head (head = 1) did not yield satisfactory imputation results. Therefore, we recommend employing the multi-head attention mechanism to improve imputation outcomes.

**Figure 7:**
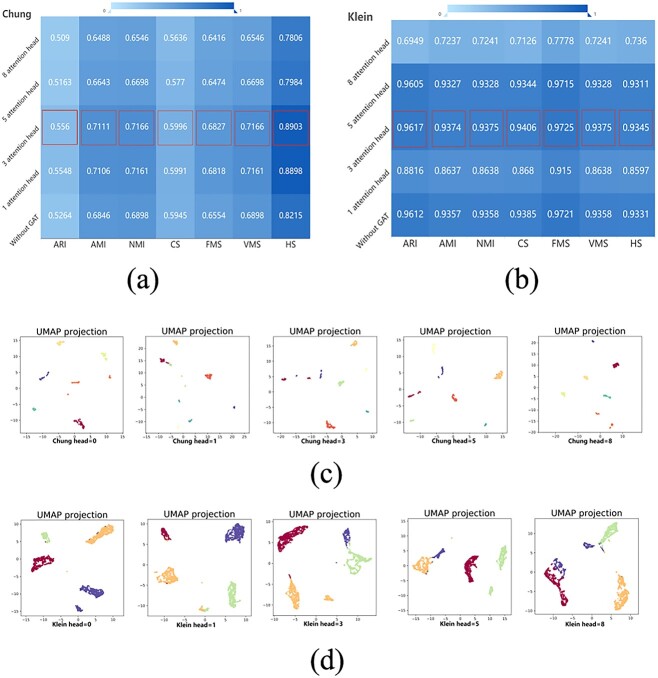
The Chung (**a**) and Klein (**b**) datasets were assessed using seven cell clustering evaluation metrics ARI, AMI, NMI, CS, FMS, VMS and HS in scMPN with and without graph attention (head = 0, head = 1, head = 3, head = 5, head = 8). The head = 3 indicates the optimal value. (**c**) and (**d**) showUMAP visualizations of the clustering results for the Chung and Klein datasets, respectively, under different conditions of graph attention.

Additionally, we evaluated the impact of graph attention on cell clustering using the Chung and Klein datasets. Figure 7(A) and (B) represent the results of eight cell clustering evaluation metrics obtained with different head values for the Chung and Klein datasets. The Chung and Klein datasets achieved the best results in the clustering tests when head = 3. Figure 7(C) and (D) visualize the clustering results obtained using the UMAP method with different head values. These visualizations provide a more intuitive understanding of how the GAT in scMPN influences the clustering outcomes of the Chung and Klein datasets.

## CONCLUSION

In the current landscape, scRNA-seq data analysis continues to evolve rapidly. Challenges persist in addressing data dimensionality reduction, gene imputation, and cell clustering. This paper introduces the scMPN model, utilizing a MLP and graph neural network to effectively analyse scRNA-seq data, encompassing gene imputation and cell clustering. ScMPN demonstrates superior performance across diverse evaluation metrics by incorporating GATs.

Using the MLP, encoder architecture reduces dimensionality and enhances denoising capabilities. The primary objective of the VGAE is to minimize the loss function, hence optimizing the output to resemble the input closely. The GAT assigns varying weights to nodes according to their individual information, improving the efficacy of sequential tasks. The scMPN model utilizes the MLP to denoise the embedding, resulting in time savings and enhanced capture of cell interactions. This, in turn, leads to better outcomes in gene imputation and cell clustering. The graph auto-encoder demonstrates enhanced efficiency in processing graph data. Moreover, when combined with the multi-head attention mechanism, it becomes possible to give distinct weights to each cell, directing attention towards the most relevant features of the data and effectively capturing the underlying connections between nodes. The performance of scMPN surpasses that of other approaches in gene imputation and cell clustering tasks.

In future work, this study will further investigate deep neural network models such as Transformer to achieve enhanced functionality in scRNA-seq data analysis. Additionally, we aim to extend the application of these models to various domains, thereby fostering the development of bio-informatics.

**Key Points**

- Single-cell RNA sequencing (scRNA-seq) is a powerful technique that allows the examination of GE at the individual cell level. This method has played a crucial role in advancing our understanding of brain cell differentiation and embryonic cell development. Moreover, scRNA-seq offers valuable information for diagnosing diseases and guiding clinical treatments.
- Analyzing data from scRNA-seq faces substantial challenges arising from its inherent variability, sparsity, and high dimensionality.
- In order to tackle these issues, as mentioned above, we propose using the scMPN model. This model integrates multi-layer perceptron, graph neural networks and attention networks to perform gene imputation and cell clustering effectively.
- In order to aid biologists in their technique selection process, several comparisons were made to assess the performance of the scMPN model in contrast to other current methodologies.
- The experiments were carried out utilizing four distinct scRNA-seq datasets: Kolodziejczy, Klein, Zeisel and Chung. These datasets are recognized for possessing reliable cell labels considered as gold standards. By employing these datasets, the assessment of the scMPN model becomes robust, providing a solid foundation for evaluating its effectiveness and suitability in real-world scenarios.

## FUNDING

This research was funded by the National Natural Science Foundation of China (No. 62262019), the Hainan Provincial Natural Science Foundation of China (No. 823RC488, No. 623RC481, No. 620RC603), Foreign Young Talents Program of the State Bureau of Foreign Experts Ministry of Science and Technology China (No. QN2023034001), the Haikou Science and Technology Plan Project of China (No. 2022-016), and Foreign Young Talents Program of the State Bureau of Foreign Experts Ministry of Science and Technology China (No. QN2023034001).

## DATA AVAILABILITY

We have passed the scMPN code to github and introduced how to use it. This is the link of scMPN on github https://github.com/fx1245/scMPN.git.
